# Healthcare-Associated Pneumonia: Don't Forget About Respiratory Viruses!

**DOI:** 10.3389/fped.2019.00168

**Published:** 2019-05-16

**Authors:** Margarita Torres-García, Brenda Berenice Pérez Méndez, José Luis Sánchez Huerta, Mónica Villa Guillén, Virydiana Rementería Vazquez, Arturo Daniel Castro Diaz, Briceida López Martinez, Almudena Laris González, Rodolfo Norberto Jiménez-Juárez, Daniela de la Rosa-Zamboni

**Affiliations:** ^1^Epidemiology Department, Federico Gómez Children's Hospital of Mexico, Mexico City, Mexico; ^2^Infectious Diseases Department, Federico Gómez Children's Hospital of Mexico, Mexico City, Mexico; ^3^Molecular Biology Department, Federico Gómez Children's Hospital of Mexico, Mexico City, Mexico; ^4^Federico Gomez Children's Hospital of Mexico, Mexico City, Mexico; ^5^Education Department, Federico Gomez Children's Hospital of Mexico, Mexico City, Mexico; ^6^Diagnostic Auxiliary Services, Federico Gomez Children's Hospital of Mexico, Mexico City, Mexico; ^7^Department of Pediatrics, National Medical Center La Raza, Infectious Diseases Hospital, Mexican Institute of Social Security, Mexico City, Mexico

**Keywords:** hospital-acquired pneumonia, viral pneumonia, respiratory viruses, nosocomial infections, healthcare-associated infections

## Abstract

**Introduction:** Healthcare-associated infections are an important cause of morbidity and mortality, are among the most common adverse events in healthcare, and of them, pneumonia is the most commonly reported. Our objective was to evaluate the incidence and clinical outcome of respiratory viruses in hospital-acquired pneumonia (HAP).

**Methods:** This was a prospective cohort study, include patients aged between 0 and 18 who fulfilled Centers for Diseases Control and Prevention (CDC) criteria for HAP. Demographic and clinical data were obtained, and a nasopharyngeal swab specimen was taken for the detection of respiratory viruses. All included patients were monitored until discharge to collect data on the need for mechanical ventilation, intensive care unit (ICU) admission, and mortality. All-cause 30-day mortality was also ascertained.

**Results:** Four thousand three hundred twenty-seven patients were followed for 42,658 patient-days and 5,150 ventilator-days. Eighty-eight patients (2.03%) met the CDC criteria for HAP, 63 patients were included, and clinical and epidemiological characteristics showed no statistically significant differences between patients with virus associated healthcare-associated pneumonia (VAHAP) and those with non-viral healthcare-associated pneumonia (NVHAP). At least one respiratory virus was detected in 65% [95% CI (53–77)] of episodes of HAP, with a single viral pathogen observed in 53.9% and coinfection with 2 viruses in 11.1% of cases. The outcome in terms of ICU admission, mechanical ventilation and the 30-day mortality did not show a significant difference between groups.

**Conclusions:** In two-thirds of the patients a respiratory virus was identified. There was no difference in mortality or the rest of the clinical outcome variables. About half of the patients required mechanical ventilation and 10% died, which emphasizes the importance of considering these pathogens in nosocomial infections, since their identification can influence the decrease in hospital costs and be taken into account in infection control policies.

## Introduction

Healthcare-associated infections are the most common adverse events in hospitals, responsible for substantial morbidity, mortality and costs (1, 2). According to a point-prevalence survey conducted in 199 hospitals in 2015, pneumonia was the most frequent healthcare-associated infection in the United States, and its prevalence has remained relatively stable in the last few years, compared with the substantial reductions in other nosocomial infections such as surgical site and urinary tract infections (3). Among healthcare-associated infections, pneumonia is the leading cause of death (4).

Studies in pediatric hospitals have described a prevalence of healthcare-associated infections between 6 and 16%, with HAP responsible for 13–18% of the total infections (5–7). Patients with HAP have an increased morbidity and mortality, especially in the case of ventilator-associated pneumonia, which is associated with a longer duration of mechanical ventilation and hospital stay, an excess crude mortality of 20–30% and an increase in hospital costs by more than USD $ 40,000 per case (8–12).

In recent years there has been an increase in the recognition of respiratory viruses as causative agents of HAP, first in outbreak reports and later in prevalence studies (13–19), the latter being made possible thanks to the implementation of Multiplex polymerase chain reaction (PCR), which can detect up to 17 respiratory viruses, with a high sensitivity and diagnostic specificity (20).

In adults, respiratory viruses have been reported in 22% of cases of HAP (21). The importance of respiratory viruses as a cause of pneumonia in hospitalized children has been highlighted by some retrospective studies and outbreak reports (13–19). However, information about the frequency and outcome of viral HAP in pediatric population is scarce, and its incidence and evolution using PCR technology has not been described in prospective studies.

The objective of this study was to describe the incidence of HAP of viral etiology in pediatric patients, and to compare its clinical characteristics and outcomes to those of patients with NVHAP.

## Methods

This was a prospective study conducted from October 15, 2016 to May 14, 2017 at the Federico Gómez Children's Hospital of Mexico, a tertiary referral hospital in Mexico City providing care to the uninsured population. It has 349 beds, 2 ICUs, one neonatal ICU and registers more than 7,000 hospitalizations per year.

Hospital-wide active surveillance was carried out by specialized nurses to identify and include patients from 0 to 18 years of age who fulfilled CDC criteria for HAP associated with or without ventilators (22). Those who were detected more than 5 days past the date of clinical onset were excluded from the study. After informed consent, demographic and clinical data were obtained, and a nasopharyngeal swab specimen was taken for the detection of respiratory viruses. Treating physicians were notified about the results.

As part of the hospital protocol, blood culture and, for those under mechanical ventilation, tracheal aspirate is taken in patients suspicious of HAP. Nevertheless, the final decision to order these or other tests, such as C-reactive protein (CRP) or procalcitonin was made by the attending physician.

All patients included were monitored until discharge to collect data on the need for mechanical ventilation, ICU admission, and mortality. All-cause 30-day mortality was also ascertained.

The study protocol was approved by the ethical committee of Federico Gomez Children's Hospital of Mexico with registration number HIM 2016-082.

### Virus Detection

The samples were processed using an RT-PCR system with microarray visualization (CLART® PneumoVir, Genomica, Spain) capable of detecting adenovirus, bocavirus, coronavirus, rhinovirus/enterovirus, influenza virus A (subtypes AH3N2, AH1N1), influenza virus B, metapneumovirus (subtypes A and B), parainfluenza virus 1, 2, 3, and 4 (subtypes A and B) and respiratory syncytial virus type A (RSV-A) and B (RSV-B), with a sensitivity of 83.3–100% depending on the virus. Patients were divided into two groups according to the results of the nasopharyngeal sample. Those patients in whom at least one respiratory virus was detected were included in the VAHAP group. Patients in whom no respiratory virus was detected were included in the NVHAP group.

### Statistical Analysis

Version 22 of the SPSS package was used. Quantitative variables were expressed as measures of central tendency and dispersion. The Kolmogorov-Smirnov normality test was performed to determine if the variables were normally distributed. The chi-squared test or Fisher's exact test were used for comparison of categorical variables and Mann-Whitney U test for quantitative data. The *p* < 0.05 was considered statistically significant. As a measure of the frequency of the disease, the incidence rate of VAHAP was estimated, with a 95% CI. We used logistic regression to estimate the risk of adverse outcomes such as ICU admission, mechanical ventilation and 30-day mortality. We also developed a Kaplan Meier analysis to address the 30-day mortality in both groups.

## Results

**Figure 1:**
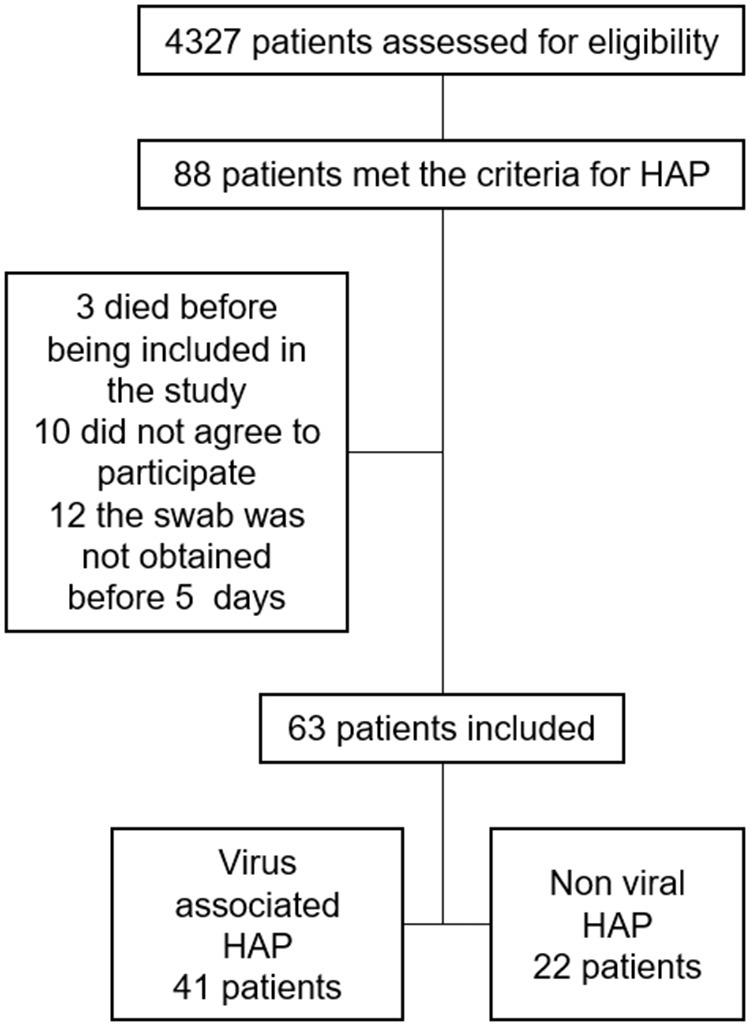
Flowchart of included patients from the surveillance.

During the study period, 4,327 patients were followed for 42,658 patient-days and 5,150 ventilator-days. Eighty-eight patients (2.03%) met the CDC criteria for HAP, for an incidence rate of 2.06/1,000 patient days [95% C. I. (1.65–2.54)], of which 9 were Ventilator associated-pneumonia, or 1.74/1,000 ventilator days. Three patients died before the procurement of nasopharyngeal samples, 10 patients did not provide consent, and for 12 patients it was not possible to obtain the nasopharyngeal sample within 5 days from the onset of clinical symptoms (Figure 1); therefore 63 patients were included and data on 1,810 patient-days were collected. Baseline epidemiological and clinical characteristics did not differ between included and excluded subjects (Table 1).

**Table 1:** Basal Characteristics of patients with hospital acquired pneumonia.

**Variable**	**Excluded *N* = 25**	**Included *N* = 63**	***P***
Age in months^*^	18 (5–80)	25 (6–103)	1^a^
Age ^**^			0.89^b^
< 28 days	3 (12)	7 (11)	
> 28 days to < 1 yo	6 (24)	20 (31)	
1 to 4 yo	9 (36)	18 (29)	
5 to 14 yo	5 (20)	15 (23)	
15 yo	2 (8)	3 (5)	
Congenital heart disease^**^	4 (16)	18 (28)	0.21^c^
Other congenital birth defects^**^	4 (16)	5 (8)	0.2^b^
Malignant neoplasia^**^	10 (40)	19 (30)	0.37^c^
Genitourinary tract disease	0	7 (11)	0.18^b^
Chronic respiratory illness	3 (12)	4 (6)	0.40^b^
Other comorbidities^**^	4 (16)	11 (18)	1^b^
Care service			0.60^c^
ICU^**^	6 (24)	12(19)	
Hospital ward	19 (76)	51 (81)	
Days of hospitalization prior to infection^*^	18 (10–30)	19 (8–36)	1^a^

* Median and IQR.

** Frequencies and percentages.

a Mann-Whitney U test was performed to determine the difference between groups.

b Exact Fisher test was performed to determine the difference between groups.

c *Pearson's chi-squared test was performed to determine the difference between groups*.

Of the included patients, 55.6% were males and the median age was 25 months old (IQR 6–103). None of the included patients referred influenza vaccination in the previous or present season. The most frequent diagnoses were malignant neoplasms (30%), congenital heart disease (28%) and malformations of the genitourinary tract (11%). Twelve cases of HAP (19%) were acquired in the ICU.

**Figure 2:**
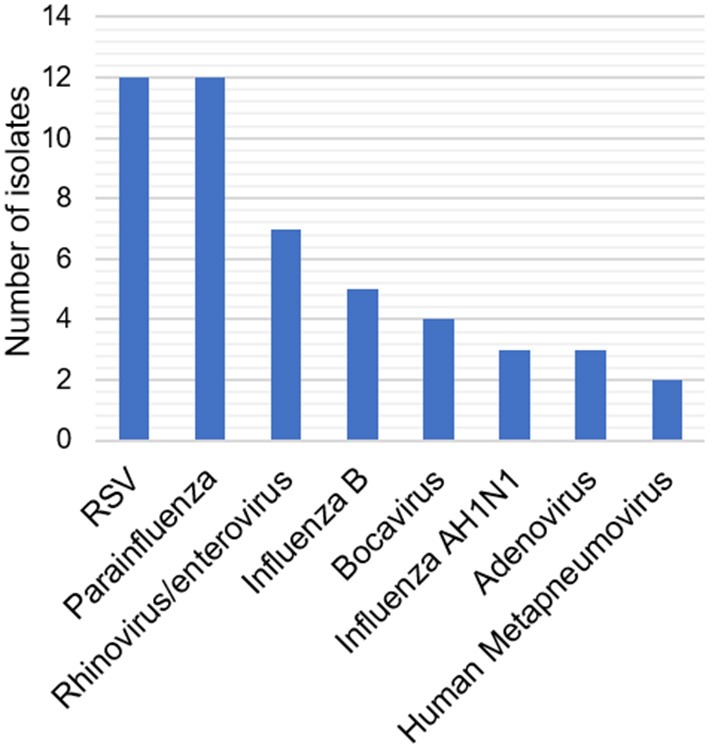
Frequency of identified respiratory viruses. The distribution of subtypes of VSR and parainfluenza virus was: RSV A 21.9%, RSV B 7.3%; Parainfluenza type 1 2.4%, type 3 19.5% ant type 4 7.3%.

At least one respiratory virus was detected in 65% [95% CI (53–77)] of HAP episodes, with a single viral pathogen observed in 53.9% and coinfection with two viruses in 11.1% of cases. The most frequently identified viruses were RSV and parainfluenza which represented 25% of the cases each, followed by influenza B and AH1N1 (17%), rhinovirus (13%), bocavirus (8%), adenovirus (6%), human metapneumovirus (4%), and enterovirus (2%) (Figure 2).

Patients with VAHAP were slightly younger [median age 21 (IQR 6–90) vs. 37 (IQR 5–126) months], had been hospitalized for a shorter period of time [median length of stay 14 (IQR 5–27) vs. 30 (IQR 13–81) days] and were more likely to have leukopenia (16.8 vs. 4.8%) than those with NVHAP. However, the strength of evidence for these differences was low (*p* > 0.05). Otherwise, epidemiological and clinical characteristics were similar between the two groups (Tables 2, 3).

**Table 2:** Characteristics of the population with VAHAP and NVHAP.

**Variable**	**NVHAP *N* = 22**	**VAHAP *N* = 41**	***P***
Age in months^*^	37 (5–126)	21 (6–90)	0.79^a^
Gender^**^			0.67
Female	9 (41)	19 (46.3)	
Male	13 (59)	22 (53.7)	
Nutritional status^**^			0.25^c^
No risk	8 (36.4)	24 (58.5)	
Overweight	1 (4.5)	1 (2.4)	
Obesity	1 (4.5)	4 (9.8)	
Malnourished	5 (22.7)	3 (7.3)	
Severely malnourished	7 (31.8)	9 (22.0)	
Length of hospital stay (days)^*^	30 (13–81)	14 (5–27)	0.84^a^
Malignant neoplasia^**^	5 (22.7)	13 (31.7)	0.45^c^
Congenital heart disease^**^	8 (36.4)	10 (24.4)	0.31^c^
Other congenital diseases^**^	4 (18.2)	2 (4.9)	0.17^b^
Genitourinary tract disease	3 (13.6)	4 (9.8)	0.68^b^
Chronic respiratory illness	1 (4.5)	3 (7.3)	1^b^
Other comorbidities^**^	2 (9.5)	9 (22.0)	0.30^b^
VAP^**^	4 (18.2)	2 (4.9)	0.17^b^
Days of antibiotic therapy^*^	10 (7–15.5)	9.5 (7–12.5)	0.45^a^

* Median and IQR.

** Frequencies and percentages.

a Mann-Whitney U test was performed to determine the difference between groups.

b Exact Fisher test was performed to determine the difference between groups.

c *Pearson's chi-squared test was performed to determine the difference between groups*.

**Table 3:** Clinical data of infection.

**Variable**	**NVHAP *N* = 22**	**VAHAP *N* = 41**	***P***
Maximum body temperature, °C^*^	38.4 (37.9–38.8)	38.4 (38–38.9)	0.817^a^
Fever^**^	15 (68.2%)	32 (78.0%)	0.33^c^
Tachypnea^**^	17 (81.0%)	27 (61.4%)	0.21^c^
${\text{O}}_{2}^{*}$ % Sat	88 (81–93)	88 (79–93)	0.67^a^
${\text{O}}_{2}^{**}$ Sat < 94%	18 (81.8%)	37 (90.2%)	0.43^b^
Leukocytosis^**^	5 (23%)	11 (26%)	0.35^c^
Leukopenia^***^	1 (4.8%)	7 (16.8%)	
Leukocytes/mm^3^*^^	13,450 (7,375–17,450)	8,800 (4,750–17,500)	0.75^a^
Neutrophils %^*^	67 (52–78)	66 (39–78)	0.74^a^
Bands %^*^	3 (1–9)	1 (0–9)	0.19^a^
Lymphocytes %^*^	22 (12–34)	19 (10–36)	0.48^a^
Monocytes %^*^	5 (3–8)	5 (3–9)	0.52^a^
Platelets/mm^3^*^^	164,000 (127,250–333,000)	231,000 (140,500–362,000)	0.88^a^
Positive cultures^**^	7 (8.7%)	8 (8.9%)	0.29^a^
Blood cultures^**^	3 (4.6%)	8 (6.9%)	0.71^b^
Positive tracheal aspirate culture^**^	4 (26.6%)	4 (21.05%)	0.42^b^

* Median and IQR.

** Frequencies and percentages.

*** < 4,000 leukocytes/mm^3^.

a Mann-Whitney U test was performed to determine the difference between groups.

b Exact Fisher test was performed to determine the difference between groups.

c *Pearson's chi-squared test was performed to determine the difference between groups*.

The median length of antibiotic prescription, although lower in the VAHAP group, did not show a significant difference between the two groups (Table 2).

Blood cultures were taken from 60 patients (95%), obtaining a total of 120 cultures. Tracheal aspirate cultures were obtained from 22 out of 33 patients with tracheal intubation (66%). Twelve patients (19%) had either a positive blood or tracheal aspirate culture (Table 3). There was no difference between the number of blood cultures or tracheal aspirates taken in the NVHAP group and the VAHAP group, (*p* > 0.2). Eight different bacterial pathogens were isolated from the patients, which were in order of frequency *Staphylococcus epidermidis* (*N* = 2), *Pseudomonas aeruginosa* (*N* = 2), *Escherichia coli* (*N* = 2), *Klebsiella pneumoniae* (*N* = 2), *Staphylococcus aureus* (*N* = 1), *Stenotrophomonas maltophilia* (*N* = 1), *Enterobacter cloacae*(*N* = 1), and *Acinetobacter baumanii* (*N* = 1).As CRP or procalcitonin were obtained in only 8 patients, these were not included in the analysis.

**Figure 3:**
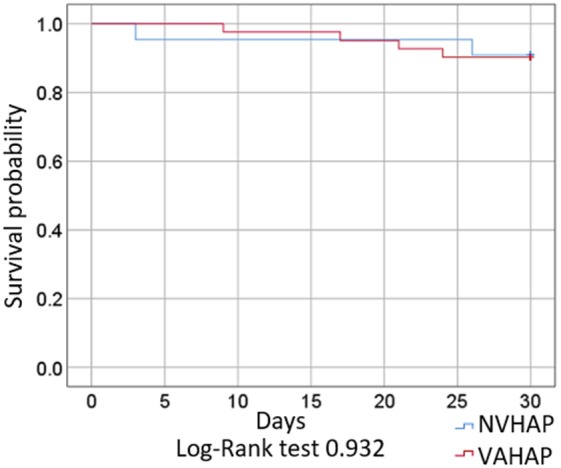
Survival of included patients with VAHAP and NVHAP.

With regards to outcome (Table 4), two thirds of patients in the NVHAP and almost half in the VAHAP group required mechanical ventilation (66.7 vs. 46.2%, RR 0.69, 95% CI 0.43–1.37). Although deaths occurred slightly earlier in the NVHAP group, the 30-day mortality rate did not show a significant difference between groups (Figure 3), nor did the risk for death differ between groups in the univariate or multivariate analysis (RR 1.07, 95% CI 0.29–5.40).

**Table 4:** Outcome of patients with VAHAP and NVHAP.

**Variable**	**NVHAP *N* = 22**	**VAHAP *N* = 41**	***P***	**RR**	**CI**
Mechanical ventilation	12 (66.7)	18 (46.2)	0.14^a^	0.69	0.43–1.37
ICU admission	3 (21.4)	13 (37.1)	0.33^b^	1.73	0.58–5.16
30-day mortality	2 (9.1)	4 (9.8)	1^2^	1.07	0.29–5.40

Data are presented as frequencies and percentages.

a Pearson's chi-squared test was performed to determine the difference between groups.

b *Exact Fisher test was performed to determine the difference between groups*.

## Discussion

This is the first prospective study that evaluates the incidence of nosocomial pneumonia of viral etiology in a pediatric setting. Importantly, at least one respiratory virus was identified in almost two thirds of patients with HAP, with a morbid outcome. Additionally, we did not find any significant difference in the clinical presentation and outcome when comparing VAHAP and NVHAP, with non-influenza respiratory viruses being predominant.

According to our results, 65% of children with HAP were positive for a respiratory virus in nasopharyngeal specimens. The importance of respiratory viruses in children with respiratory infections was discussed first by Wenzel in 1977 (23) and in nosocomial pneumonia in 1980 by Valenti et al. In this retrospective study, at least one respiratory virus was identified in 20% of pediatric patients with nosocomial pneumonia (24). Recently, a retrospective analysis carried out in a single center in Washington identified at least one respiratory virus in 22% of adults with non-ventilated HAP (21). The higher frequency of respiratory viruses found in our study could be due to the systematic active surveillance of nosocomial pneumonia and, compared with the study by Valenti et al., to the availability of molecular methods that increased the sensitivity for the detection of respiratory viruses in our study.

We did not identify any epidemiologic or clinical characteristics that could reliably distinguish pediatric patients with a respiratory virus infection among those with HAP. However, this might be explained by the low case numbers in our population and should be further explored in larger studies.

The median length of stay at the time of onset of pneumonia was shorter in patients with VAHAP than among those with NVHAP, but there was a wide overlap in the distribution of the variable.

The high incidence of morbidity outcomes (admission to intensive care, mechanical ventilation) and death in both groups suggests that, regardless of the participation of respiratory viruses, the outcome of HAP in this population is adverse. Two deaths were attributable to HAP according to the attending physician. Of those two cases, adenovirus was detected in one of them and the other died with methicillin-resistant *Staphylococcus aureus* septicemia 15 days after the diagnosis of influenza. Due to the well-known association of *Staphylococcus aureus* infection with a previous episode of influenza (25), we can assume that respiratory viruses also contributed to the death of this patient.

One-third of patients with VAHAP in our study required admission to the ICU. Choi in 2012 (26) reported that more than a third of adults (34%) with HAP that required admission to intensive therapy presented at least one type of respiratory virus. In the previously mentioned study in adults with non-ventilated HAP in Washington, Shorr et al. (21) reported that the outcomes were similar between patients with viral and bacterial pneumonia. Almost half of the patients with VAHAP in our study required mechanical ventilation, which points to the severity of nosocomial pneumonia associated with respiratory viruses. However, our study was not powered to find differences in outcomes between groups.

Finally, respiratory viruses other than influenza comprised 80% of cases of VAHAP and one half of all cases of nosocomial pneumonia. This coincides with the findings of previous studies, in which the main viruses identified in patients with HAP were rhinovirus, respiratory syncytial virus, parainfluenza and coronavirus (21, 23, 27–29). This highlights the relevance of infection control measures in preventing the spread of respiratory viruses from patients, caregivers and healthcare personnel, focusing not only on individuals with influenza-like illness, but also those with common colds or other respiratory symptoms. It also highlights the importance of developing effective vaccines and drug therapies for respiratory viruses other than influenza, given the current scarcity of preventive and therapeutic interventions. Despite the lack of specific treatment for most cases of VAHAP, routine identification of respiratory viruses in these patients could be useful to support a decision of withholding or stopping antibiotics in certain cases with no evidence of bacterial infection, potentially reducing adverse effects, costs and drug resistance.

The present study presents certain limitations. Only 71% of patients with HAP were included in the study. However, when comparing the baseline characteristics of these subjects with those who were not included, no statistically significant differences were observed (Table 1), which supports the representativeness of the sample. On the other hand, tests such as broncho alveolar lavage, or broncho alveolar brushing, which could increase the sensitivity to bacteria, are scarcely performed because they are considered invasive and not essential for patient care. Although difficult to overcome, this could be an important limitation because we were not able to assure that the infection was solely due to a viral etiology. In addition, we did not interfere in the management of the patients and, therefore, the frequency of these tests and others, such as PCT or CRP, was not altered during the study. More studies with the routine use of PCT or other markers of bacterial infections are needed. Another issue that should be clarified in further studies is the clinical implications of each type of respiratory viruses since some of them (i.e., influenza, SRV) are considered clinically relevant more often than others, such as parainfluenza and some types of rhinovirus, and our study was not powered to detect such differences. Lastly the study was conducted in a tertiary care hospital with a non-homogeneous sample of patients in which two thirds of the participants had at least one known risk factor for viral pneumonia, such as chronic lung disease (67%), congenital heart disease (29%), and neoplasia (27%), so the results obtained should be taken into account in this context and not necessarily generalized to all pediatric patients with HAP.

In conclusion, we found a high prevalence of respiratory virus infection among children with HAP, therefore it is necessary to implement preventive measures to reduce the burden of these agents and consider strategies to optimize antibiotic use in this group of patients. The development of new vaccines or drugs for prevention and treatment of respiratory viruses other than influenza would potentially be of major benefit for children with HAP. Larger studies in different hospital settings are needed to better define the clinical spectrum of viral HAP.

## Data Availability

All datasets generated for this study are included in the manuscript and/or the supplementary files.

## Ethics Statement

This study was carried out in accordance with the recommendations of Norma Oficial Mexicana NOM-012-SSA3-2012, and committees of research, ethics and biosafety of the Hospital Infantil de México Federico Gómez with written informed consent from all subjects. All subjects gave written informed consent in accordance with the Declaration of Helsinki. The protocol was approved by the committees of research, ethics and biosafety of the Hospital Infantil de México Federico Gómez.

## Author Contributions

MT-G and DR-Z contributed to the conception and design of study. MT-G, BP, VR, and DR-Z contributed to the acquisition of data. MT-G, BP, JS, MV, RJ-J, AL, and DR-Z contributed to the analysis and/or interpretation of data. MT-G, BP, VR, AC, BL, AL, and DR-Z drafted the manuscript. MT-G, JS, MV, BL, AL, RJ-J, and DR-Z contributed to the critical revision of the manuscript for important intellectual content. MT-G, BP, JS, MV, VR, AC, BL, AL, RJ-J, and DR-Z approved the final version of this manuscript.

### Conflict of Interest Statement

The authors declare that the research was conducted in the absence of any commercial or financial relationships that could be construed as a potential conflict of interest.

## Abbreviations

HAP: Hospital-acquired pneumonia
ICU: Intensive care unit
VAHAP: Virus associated hospital-acquired pneumonia
NVHAP: Non-viral hospital-acquired pneumonia
RT-PCR: Reverse transcription polymerase chain reaction
CDC: Centers for Diseases Control and prevention
CRP: C-reactive protein
RSV-A: respiratory syncytial virus type A
RSV-B: respiratory syncytial virus type and B.

## References

[B1] Leape LL Brennan TA Laird N Lawthers AG Localio AR Barnes BA. The nature of adverse events in hospitalized patients: results of the Harvard Medical Practice Study II. N Engl J Med. (1991) 324:377–84. 10.1056/NEJM199102073240605 1824793

[B2] Cardo D Dennehy PH Halverson P Fishman N Kohn M Murphy CL. Moving toward elimination of healthcare-associated infections: **a** call to action. Am J Infect Control. (2010) 38:671–5. 10.1016/j.ajic.2010.09.001 21058460

[B3] Magill SS O'Leary E Janelle SJ Thompson DL Dumyati G Nadle J. Changes in Prevalence of Health Care-Associated Infections in U.S. Hospitals. N Engl J Med. (2018) 379:1732–44. 10.1056/NEJMoa1801550 30380384 PMC7978499

[B4] Glied S Cohen B Liu J Neidell M Larson E. Trends in mortality, length of stay, and hospital charges associated with health care-associated infections, 2006-2012. Am J Infect Control. (2016) 44:983–9. 10.1016/j.ajic.2016.03.010 27207157 PMC5011005

[B5] Mühlemann K Franzini C Aebi C Berger C Nadal D Stähelin J. Prevalence of nosocomial infections in Swiss children's hospitals. Infect Control Hosp Epidemiol. (2004) 25:765–71. 10.1086/502474 15484802

[B6] Rutledge-Taylor K Matlow A Gravel D Embree J Le Saux N Johnston L. A point prevalence survey of health care-associated infections in Canadian pediatric inpatients. Am J Infect Control. (2012) 40:491–6. 10.1016/j.ajic.2011.08.008 22078941

[B7] Zamudio-Lugo I Espinosa-Vital GJ Rodríguez-Sing R Gómez-González CJ Miranda-Novales MG Infecciones nosocomiales. Tendencia durante 12 a-os en un hospital pediatrico. Rev Med Inst Mex Seguro Soc. (2014) 52:38–43. 24983553

[B8] Rello J Ollendorf DA Oster G. Epidemiology and outcomes of ventilator-associated pneumonia in a large US database. Chest. (2002) 122:2115–21. 10.1378/chest.122.6.2115 12475855

[B9] Hu B Tao L Rosenthal VD Liu K Yun Y Suo Y. Device-associated infection rates, device use, length of stay, and mortality in intensive care units of 4 Chinese hospitals: International nosocomial control consortium findings. Am J Infect Control. (2013) 41:301–6. 10.1016/j.ajic.2012.03.037 23040491

[B10] Patra P Jayashree M Singhi S Ray P Saxena A. Nosocomial pneumonia in a pediatric intensive care unit. Indian Pediatr. (2007) 44:511–8. 17939179

[B11] Al-Mousa HH Omar AA Rosenthal VD Salama MF Aly NY El-Dossoky Noweir M. Device-associated infection rates, bacterial resistance, length of stay, and mortality in Kuwait: International nosocomial infection consortium findings. Am J Infect Control. (2016) 44:444–9. 10.1016/j.ajic.2015.10.031 26775929

[B12] Fagon JY Chastre J Hance AJ Montravers P Novara A Gibert C. Nosocomial pneumonia in ventilated patients: a cohort study evaluating attributable mortality and hospital stay. Am J Med. (1993) 94:281–8. 10.1016/0002-9343(93)90060-3 8452152

[B13] Morrow BM Argent AC. Ventilator-associated pneumonia in a paediatric intensive care unit in a developing country with high HIV prevalence. J Paediatr Child Health. (2009) 45:104–11. 10.1111/j.1440-1754.2008.01437.x 19210603

[B14] Hall CB. Nosocomial viral respiratory infections: perennial weeds on pediatric wards. Am J Med. (1980) 70:670–6. 10.1016/0002-9343(81)90594-5 6259940

[B15] Spaeder MC Custer JW Bembea MM Aganga DO Song X Scafidi S. A multicenter outcomes analysis of children with severe viral respiratory infection due to human metapneumovirus. Pediatr Crit Care Med. (2013) 14:268–72. 10.1097/PCC.0b013e3182720fc7 23392374

[B16] van de Pol AC Rossen JWA Wolfs TFW Breteler EK Kimpen JLL Loon AM Van. Transmission of respiratory syncytial virus at the paediatric intensive-care unit : a prospective study using real-time PCR. Eur Soc Clin Infect Dis. (2009) 16:488–90. 10.1111/j.1469-0691.2009.02854.x 19523052

[B17] Durigon GS Oliveira DBL Vollet SB Storni JG Felício MCC Finelli C. Hospital-acquired human bocavirus in infants. J Hosp Infect. (2017) 76:171–3. 10.1016/j.jhin.2010.04.028 20619493 PMC7114665

[B18] Piralla A Percivalle E Di Cesare-Merlone A Locatelli F Gerna G. Multicluster nosocomial outbreak of parainfluenza virus type 3 infection in a pediatric oncohematology unit: a phylogenetic study. Haematologica. (2009) 94:833–9. 10.3324/haematol.2008.003319 19377073 PMC2688575

[B19] Kim S Sung H Im HJ Hong SJ Kim MN. Molecular epidemiological investigation of a nosocomial outbreak of human metapneumovirus infection in a pediatric hemato-oncology patient population. J Clin Microbiol. (2009) 47:1221–4. 10.1128/JCM.01959-08 19213698 PMC2668301

[B20] Mahony JB. Detection of respiratory viruses by molecular methods. Clin Microbiol Rev. (2008) 21:716–47. 10.1128/CMR.00037-07 18854489 PMC2570148

[B21] Shorr AF Zilberberg MD Micek ST Kollef MH. Viruses are prevalent in non-ventilated hospital-acquired pneumonia. Respir Med. (2017) 122:76–80. 10.1016/j.rmed.2016.11.023 27993295 PMC7135153

[B22] Centers for Diseases Control and Prevention (CDC) Pneumonia (Ventilator-Associated [VAP] and Non-ventilator-Associated Pneumonia [PNEU]) Event. (2019). p. 1–15. Available online at: https://www.cdc.gov/nhsn/PDFs/pscManual/6pscVAPcurrent.pdf (acessed January 9, 2019).

[B23] Wenzel RP Chandler Deal E Hendley O. Hospital-Acquired Viral Respiratory Illness on a Pediatric Ward. Pediatrics. (1977) 60:367–71. 197478

[B24] Valenti W Menegus M Hall C Pincus P Douglas RJ. Nosocomial viral infections: I. Epidemiology and significance. Infect Control. (1980) 1:33–7. 10.1017/S0195941700052371 6274823

[B25] Tong SYC Davis JS Eichenberger E Holland TL Fowler VG. Staphylococcus aureus infections: epidemiology, pathophysiology, clinical manifestations, and management. Clin Microbiol Rev. (2015) 28:603–61. 10.1128/CMR.00134-14 26016486 PMC4451395

[B26] Choi SH Hong SB Ko GB Lee Y Park HJ Park SY. Viral infection in patients with severe pneumonia requiring intensive care unit admission. Am J Respir Crit Care Med. (2012) 186:325–32. 10.1164/rccm.201112-2240OC 22700859

[B27] Choi HS Kim MN Sung H Lee JY Park HY Kwak SH. Laboratory-based surveillance of hospital-acquired respiratory virus infection in a tertiary care hospital. Am J Infect Control. (2017) 45:e45–7. 10.1016/j.ajic.2017.01.009 28214160 PMC7124227

[B28] Chow EJ Mermel LA. Hospital-acquired respiratory viral infections: Incidence, morbidity, and mortality in pediatric and adult patients. Open Forum Infect Dis. (2017) 4:1–6. 10.1093/ofid/ofx006 28480279 PMC5414085

[B29] Hong HL Hong SB Ko GB Huh JW Sung H Do KH. Viral infection is not uncommon in adult patients with severe hospital-acquired pneumonia. PLoS ONE. (2014) 9:e95865. 10.1371/journal.pone.0095865 24752070 PMC3994115

